# Evidence of Mediation by Depressive Symptoms in the Association Between Frailty and Cognitive Decline in Older Adults

**DOI:** 10.1093/geroni/igaa057.541

**Published:** 2020-12-16

**Authors:** Nicholas Resciniti, Matthew Lohman, Anwar Merchant

**Affiliations:** University of South Carolina, Columbia, South Carolina, United States

## Abstract

Frailty and pre-frailty have been shown to predict cognitive decline in older adults; however, knowledge about mediating pathways in this association is lacking. This study aims to assess if depressive symptoms mediate the relationship between frailty and cognitive function. Frailty status, cognitive function scores, and depressive symptoms were measured in 4,672 community-dwelling US adults ≥65 and older from the Health and Retirement Study from 2006-2010. Fried’s frailty phenotype criteria (weakness, slowness, physical inactivity, low weight, and exhaustion) were used to categorize individuals as frail (3-5 criteria), pre-frail (1-2 criteria) and robust (0 criteria). Memory recall and global mental status (from 0-35) was used to determine cognitive function. The CES-D was used to assess depressive symptoms. A causal mediation analysis was performed to estimate the direct effect (DE), the direct effect (DE), and the indirect effect (IE). Both frailty (TE: β=-1.22; 95% CI: -1.75, -0.68) and pre-frailty (TE: β=-0.52; 95% CI: -0.86, -0.18) were found to be associated with lower cognitive scores, after adjusting for confounders. There was significant but small IE between frailty status and declining cognitive scores mediated by depressive symptoms [frailty: β=-0.09 (95% CI: -0.14, -0.03); pre-frailty: β = -0.03 (95% CI: -0.06, -0.01). Additionally, the DE was significant for frailty (β=-1.13; 95% CI: -1.67, -0.59) and pre-frailty (β=-0.49 95% CI: -0.82, -0.15). This study provides evidence that depressive symptoms may mediate the association between frailty and cognitive decline. The results suggest possible intervenable pathways for preventing or delaying cognitive decline.

